# Mandarin Fish (Siniperca chuatsi) p53 Regulates Glutaminolysis Induced by Virus via the p53/miR145-5p/c-Myc Pathway in Chinese Perch Brain Cells

**DOI:** 10.1128/spectrum.02727-21

**Published:** 2022-03-14

**Authors:** Caimei Ye, Shangui Liu, Ningqiu Li, Shaozhi Zuo, Yinjie Niu, Qiang Lin, Hongru Liang, Xia Luo, Xiaozhe Fu

**Affiliations:** a Pearl River Fishery Research Institute, Chinese Academy of Fishery Sciences, Key Laboratory of Fishery Drug Development, Ministry of Agriculture and Rural Affairs, Key Laboratory of Aquatic Animal Immune Technology, Guangzhou, China; University of Manitoba

**Keywords:** *Siniperca chuatsi*, Sc-p53, glutaminolysis, p53/miR145-5p/c-Myc, ISKNV, SCRV

## Abstract

p53, as an important tumor suppressor protein, has recently been implicated in host antiviral defense. The present study found that the expression of mandarin fish (Siniperca chuatsi) p53 (Sc-p53) was negatively associated with infectious spleen and kidney necrosis virus (ISKNV) and *Siniperca chuatsi* rhabdovirus (SCRV) proliferation as well as the expression of glutaminase 1 (GLS1) and glutaminolysis pathway-related enzymes glutamate dehydrogenase (GDH) and isocitrate dehydrogenase 2 (IDH2). This indicated that Sc-p53 inhibited the replication and proliferation of ISKNV and SCRV by negatively regulating the glutaminolysis pathway. Moreover, it was confirmed that miR145-5p could inhibit c-Myc expression by targeting the 3′ untranslated region (UTR). Sc-p53 could bind to the miR145-5p promoter region to promote its expression and to further inhibit the expression of c-Myc. The expression of c-Myc was proved to be positively correlated with the expression of GLS1 as well. All these suggested a negative relationship between the Sc-p53/miR145-5p/c-Myc pathway and GLS1 expression and glutaminolysis. However, it was found that after ISKNV and SCRV infection, the expressions of Sc-p53, miR145-5p, c-Myc, and GLS1 were all significantly upregulated, which did not match the pattern in normal cells. Based on the results, it was suggested that ISKNV and SCRV infection altered the Sc-p53/miR145-5p/c-Myc pathway. All of above results will provide potential targets for the development of new therapeutic strategies against ISKNV and SCRV.

**IMPORTANCE** Infectious spleen and kidney necrosis virus (ISKNV) and *Siniperca chuatsi* rhabdovirus (SCRV) as major causative agents have caused a serious threat to the mandarin fish farming industry (J.-J. Tao, J.-F. Gui, and Q.-Y. Zhang, Aquaculture 262:1–9, 2007, https://doi.org/10.1016/j.aquaculture.2006.09.030). Viruses have evolved the strategy to shape host-cell metabolism for their replication (S. K. Thaker, J. Ch’ng, and H. R. Christofk, BMC Biol 17:59, 2019, https://doi.org/10.1186/s12915-019-0678-9). Our previous studies showed that ISKNV replication induced glutamine metabolism reprogramming and that glutaminolysis was required for efficient replication of ISKNV and SCRV. In the present study, the mechanistic link between the p53/miR145-5p/c-Myc pathway and glutaminolysis in the Chinese perch brain (CPB) cells was provided, which will provide novel insights into ISKNV and SCRV pathogenesis and antiviral treatment strategies.

## INTRODUCTION

The p53 tumor suppressor protein is a major host cellular response protein, which can be activated upon various stress signals, including DNA damage and oncogene activation, and orchestrates a plethora of downstream responses, such as DNA repair, cell cycle arrest, senescence, metabolism, and cell death (1–3). A previous study also showed that p53 could be activated by viral infections, which in turn induces apoptosis of the infected cells to limit viral replication (4). However, p53 appears to have both positive and negative effects on various viral infections. The replication of some viruses, including influenza A virus (IAV) (5), porcine epidemic diarrhea virus (PEDV) (6), and human immunodeficiency virus type 1 (HIV-1) (7), was negatively correlated with the expression of p53. Conversely, p53 is required for efficient viral replication of other viruses, including herpes simplex virus 1 (HSV-1) (8), human cytomegalovirus (HCMV) (9), and pseudorabies virus (PRV) (10).

Many studies reported that glutamine (Gln) is an essential nutrient for the proliferation of viruses and that glutaminolysis plays a critical role in viral replication (11–13). In glutaminolysis, glutamine is converted to glutamate catalyzed by glutaminase 1 (GLS1) and then catabolized to α-ketoglutarate (α-KG) through the glutamate dehydrogenase (GDH) (14). Therefore, the glutamine catabolism pathway is initiated by GLS1 (15). The oncogene c-Myc is a major regulator of cell proliferation, which regulates the expression of GLS1 through miRNA-23a and miRNA-23b (16, 17). Moreover, it has been described previously that p53 is involved in glutamine metabolism (18, 19). Ragimov et al. found that p53 could significantly inhibit c-Myc expression, suggesting there must be a negative regulatory pathway between p53 and c-Myc (20). Then, Sachdeva et al. confirmed that p53 directly interacted with the promoter region of miR145 to inhibit c-Myc expression through miR145 (21). miR145 is a precursor of miR145-3p and miR145-5p, and miR145-5p has been shown to act as a tumor suppressor in different tumor types (22, 23).

Our previous study reported that expression of p53 was upregulated in mandarin fish after viral infection, suggesting that p53 plays a critical role in antiviral responses (24). In addition, glutaminolysis was a necessary condition for efficient replication of infectious spleen and kidney necrosis virus (ISKNV) and Siniperca chuatsi rhabdovirus (SCRV) (12, 25). Accordingly, we inferred that mandarin fish p53 (Sc-p53) could negatively regulate GLS1 to affect glutaminolysis by negatively regulating c-Myc via the Sc-p53/miR145-5p/c-Myc pathway. In this study, we investigated the effects of Sc-p53 on ISKNV and SCRV replication; the expression levels of three key enzymes in the glutaminolysis pathway were determined after inhibiting or activating Sc-p53 expression. Furthermore, the promoter region of the miR145-5p gene was cloned by PCR and the full length of c-Myc cDNA was cloned by the RACE (rapid amplification of cDNA ends) method. The existence of the Sc-p53/miR145-5p/c-Myc pathway was confirmed by dual-luciferase reporter assay, and its response to ISKNV and SCRV infection was further studied. These findings shed some light on the antiviral mechanism of p53 and provide potential therapeutic targets for antiviral drugs in fish.

## RESULTS

### Effects of Sc-p53 on ISKNV and SCRV replication.

**FIG 1:**
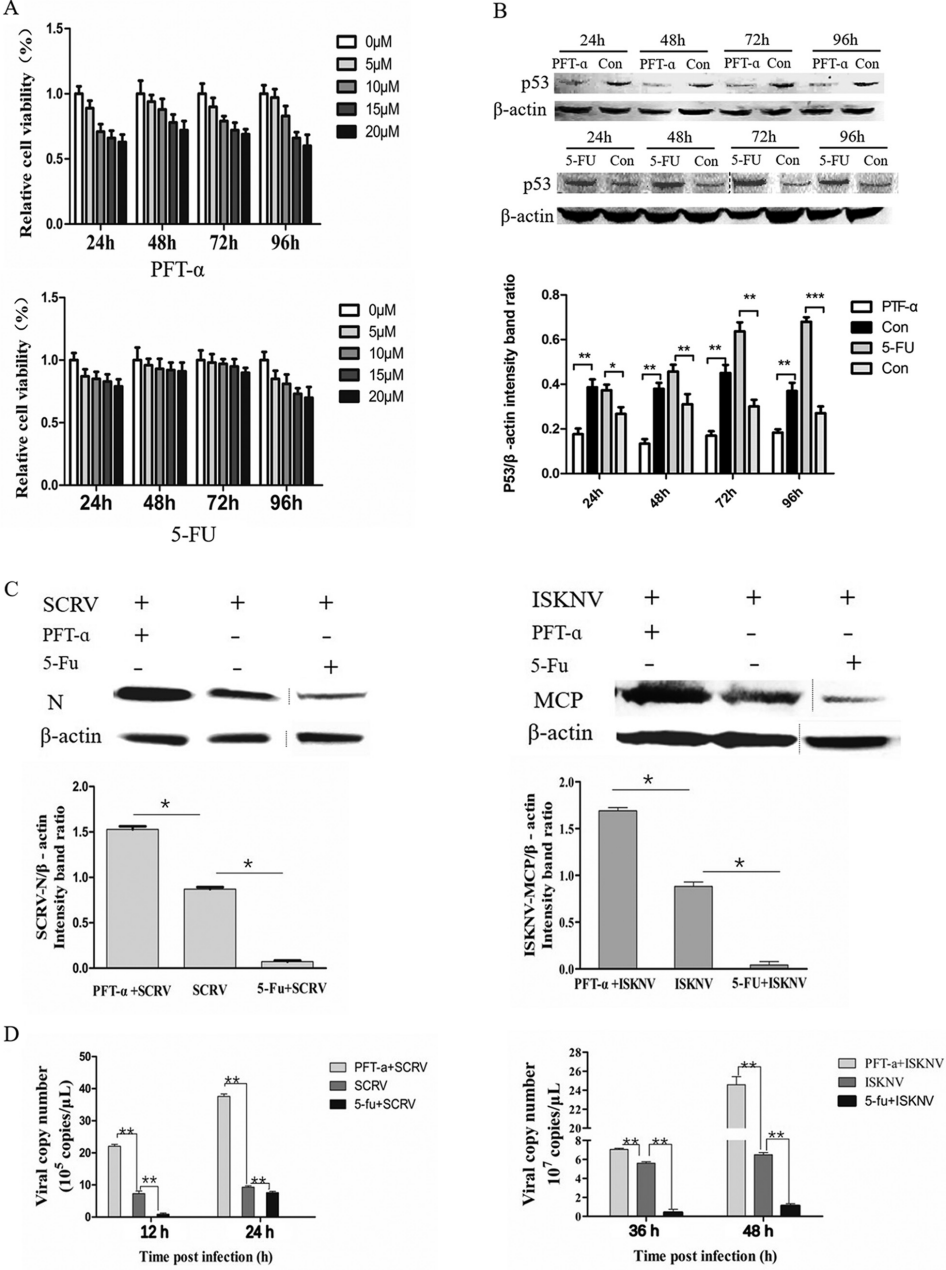
Effects of regulating Sc-p53 on ISKNV and SCRV replication. (A) Cell viability measured by MTS assay in CPB cells treated with PFT-α or 5-Fu. (B) Western blotting of p53 protein. (C) Viral protein in CPB cells treated with PFT-α or 5-Fu. β-Actin was used as a control. (D) Viral copy numbers were measured by qRT-PCR in CPB cells treated with PFT-α or 5-Fu. Asterisks indicate significant differences from the control group (*, *P* < 0.05; **, *P* < 0.01; ***, *P* < 0.001).

The MTS [3-(4,5-dimethylthiazol-2-yl)-5-(3-carboxymethoxyphenyl)-2-(4-sulfophenyl)-2H-tetrazolium] results showed that when Chinse perch brain (CPB) cells were inoculated with 10 μM pifithrin-α (PFT-α) or 5-fluorouracil (5-Fu), the cell viability was more than 80% (Fig. 1A). The Sc-p53 protein level was significantly decreased/increased in CPB cells treated with PFT-α/5-Fu (Fig. 1B). These results indicated that PFT-α and 5-Fu could effectively inhibit and activate the expression of Sc-p53, respectively, when both working concentrations were 10 μM.

To verify the effects of Sc-p53 on ISKNV and SCRV replication, CPB cells were infected with ISKNV or SCRV after treatment with PFT-α or 5-Fu for 2 h, respectively. The results showed that SCRV-N protein and ISKNV-MCP (major capsid protein) levels in the 5-Fu-treated group were significantly lower than those in the PFT-α-treated group (Fig. 1C). Furthermore, viral copies of SCRV and ISKNV were significantly increased in the PFT-α-treated cells but remarkably decreased in the 5-Fu-treated cells (Fig. 1D), which was consistent with the results of viral protein level. These results suggested that Sc-p53 could inhibit the replication of ISKNV and SCRV.

### Sc-p53 can regulate the glutaminolysis pathway.

**FIG 2:**
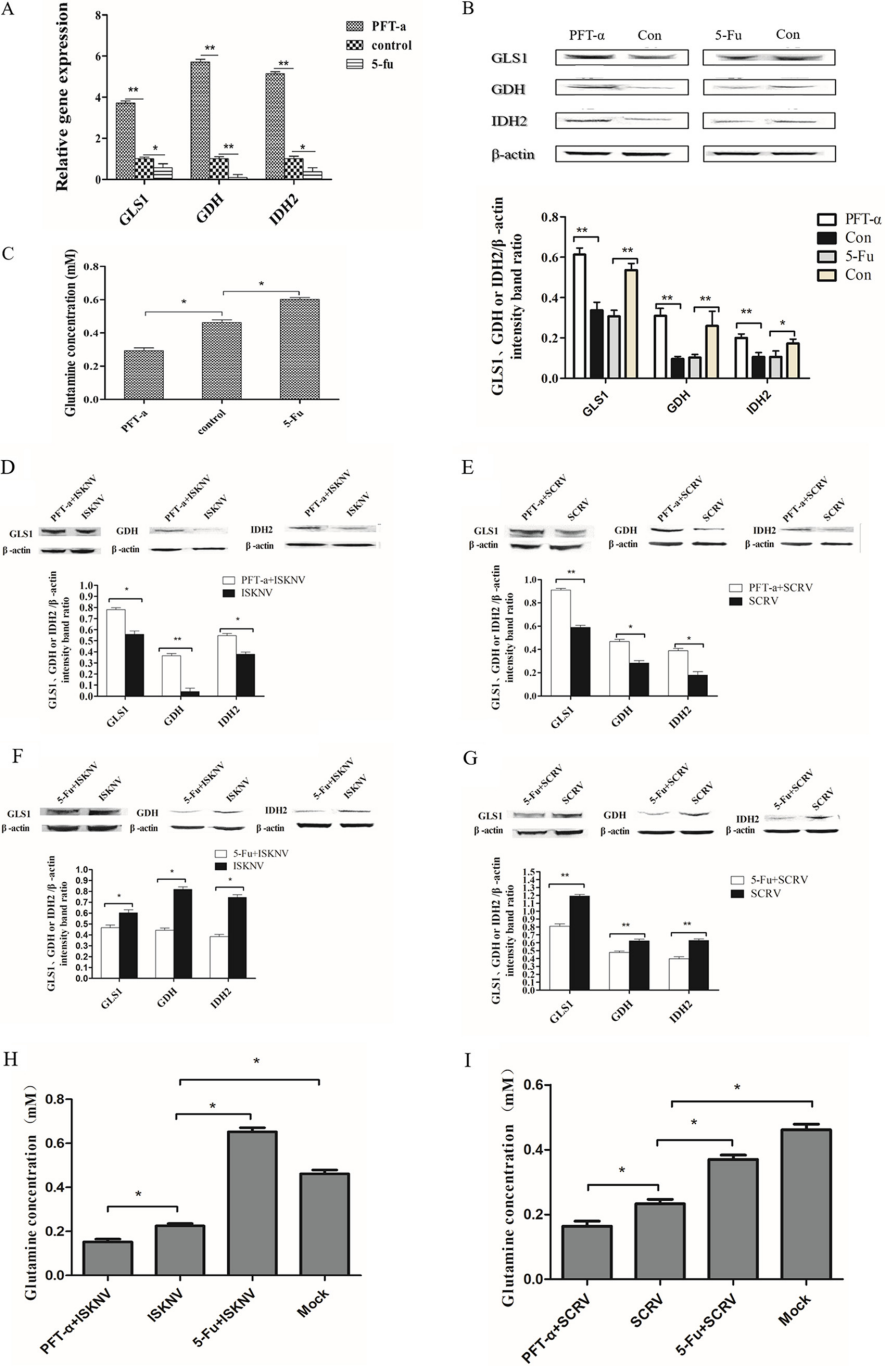
Effects of regulating Sc-p53 on glutaminolysis pathway. (A and B) The mRNA expression (A) and protein expression (B) of GLS1, GDH, and IDH2 in CPB cells treated with PFT-α or 5-Fu by qRT-PCR or Western blotting. (C) Glutamine concentration in CPB cells treated with PFT-α or 5-Fu. (D and F) Protein expression of GLS1, GDH, and IDH2 after ISKNV infection in CPB cells treated with PFT-α or 5-Fu by Western blotting. (E and G) Protein expression of GLS1, GDH, and IDH2 after SCRV infection in CPB cells treated with PFT-α or 5-Fu by Western blotting. (H and I) Glutamine concentration after ISKNV and SCRV infection in CPB cells treated with PFT-α or 5-Fu. β-Actin was used as a control for qRT-PCR and Western blotting analysis. Asterisks indicate the significant differences from the control group (*, *P* < 0.05; **, *P* < 0.01).

To evaluate whether Sc-p53 could affect glutaminolysis, the expression levels of three key enzymes (GLS1, GDH, and isocitrate dehydrogenase 2 [IDH2]) related to the glutaminolysis pathway in the cells were detected after treatment with p53 inhibitor and activator, respectively. As shown in Fig. 2A, the mRNA expression levels of GLS1, GDH, and IDH2 were significantly upregulated in PFT-α-treated cells compared to control cells. In contrast, the mRNA expression levels of GLS1, GDH, and IDH2 were significantly downregulated in 5-Fu-treated cells compared to control cells. Western blotting results showed that the protein expression levels were consistent with the transcription levels (Fig. 2B). Moreover, changes of intracellular glutamine concentration in CPB cells treated with PFT-α or 5-FU were detected. The results showed that compared with control cells, the glutamine concentration was significantly decreased in PFT-α-treated cells and significantly increased in 5-Fu-treated cells (Fig. 2C). These results indicated that Sc-p53 could negatively regulate the glutaminolysis pathway by negatively regulating GLS1.

In addition, Western blotting results showed that Sc-p53 still negatively regulated the expression of GLS1, GDH, and IDH2 after ISKNV and SCRV infection (Fig. 2D to G). The intracellular glutamine consumption of CPB cells infected with ISKNV or SCRV was also detected. As shown in Fig. 2H and I, the glutamine concentration was significantly decreased in PFT-α-treated cells and significantly increased in 5-Fu-treated cells compared with control cells. The results further confirmed that Sc-p53 still negatively regulated the glutaminolysis pathway after ISKNV and SCRV infection.

### Relationship between Sc-p53, miR145-5p and c-Myc. (i) Sc-p53 binding to the miR145-5p promoter.

**FIG 3:**
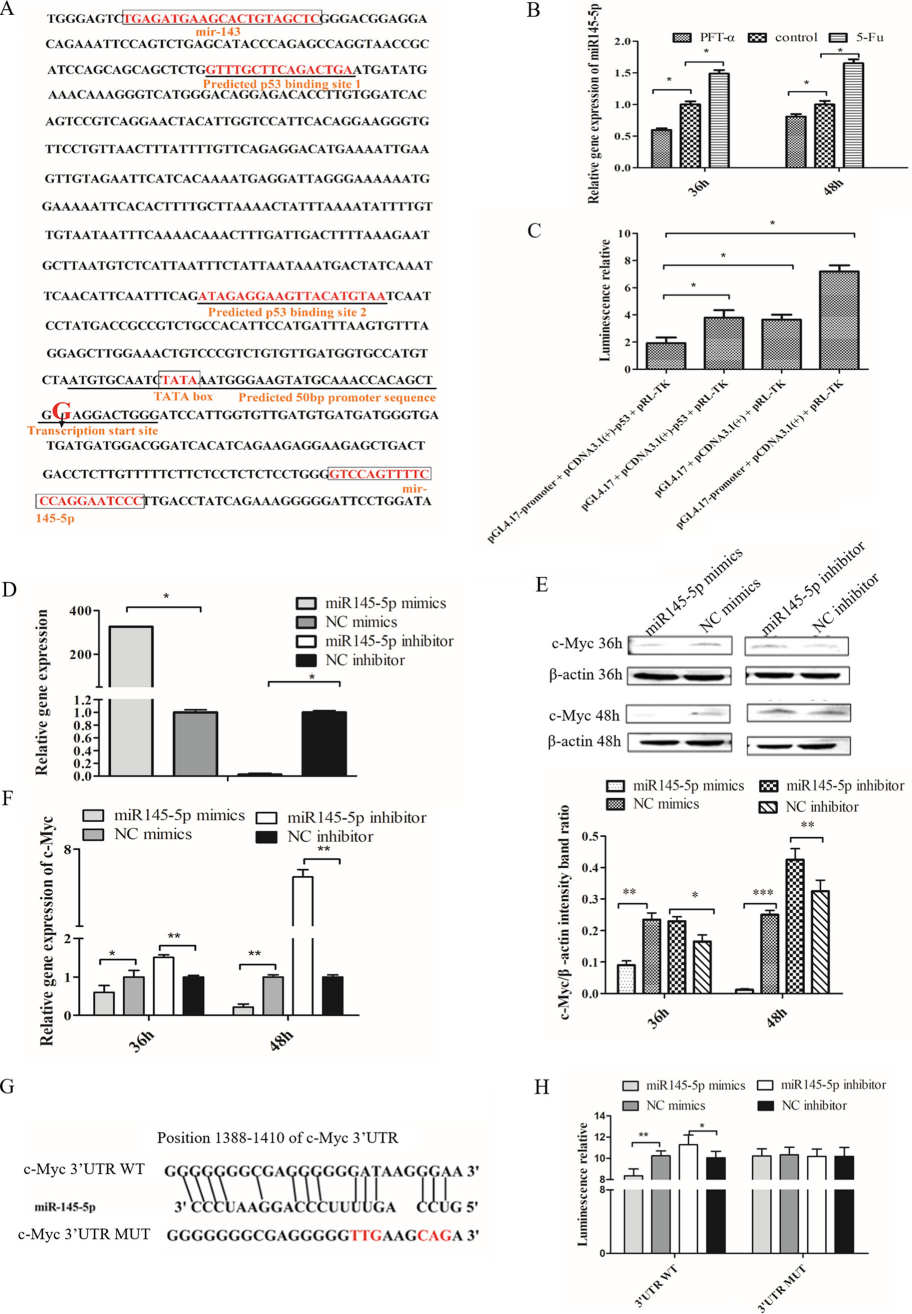
Sc-p53 targeted to the miR145-5p promoter, and miR145-5p targeted to the 3′ UTR of c-Myc. (A) Sequence of the predicted p53 binding sites at the promoter of miR145-5p. (B) mRNA expression of miR145-5p in CPB cells treated with PFT-α or 5-Fu by qRT-PCR. (C) Relative luciferase activities of p53 expression vector and miR145-5p promoter reporter vector were detected by using a dual-luciferase reporter assay. (D) mRNA expression of miR145-5p in CPB cells treated with mimics or inhibitor of miR154-5p by qRT-PCR. (E and F) Protein expression (E) and mRNA expression (F) of c-Myc in CPB cells treated with mimics or inhibitor of miR154-5p by qRT-PCR or Western blotting. (G) Alignment of miR145-5p with the predicted target sequences in the 3′ UTR of c-Myc mRNA. (H) Relative luciferase activities of WT and MUT c-Myc reporters were detected by using the dual-luciferase reporter assay. β-Actin was used as a control for qRT-PCR and Western blotting analysis. Asterisks indicate significant differences from the control group (*, *P* < 0.05; **, *P* < 0.01; ***, *P* < 0.001).

A 747-bp DNA sequence containing miR145-5p promoter sequence was amplified by PCR and confirmed by sequence alignment software Vector NTI 8.0, respectively. Promoter prediction was carried out through Promoter 2.0 and the Neural Network Promoter website. As shown in Fig. 3A, the sequence has a 50-bp promoter fragment containing a TATA box and a transcription start site, and two p53 binding sites. This suggested that Sc-p53 probably binds to the miR145-5p promoter region. Furthermore, the expression of miR145-5p was significantly decreased after treatment with PFT-α and significantly increased after treatment with 5-Fu (Fig. 3B). Subsequently, we investigated the targeting relationship between Sc-p53 and the miR145-5p promoter region by the dual-luciferase report system. The results showed that compared with the control group, the luciferase activity in the experimental group [cotransfected with pGL4.17-promoter plasmid and pCDNA3.1(+)-p53 plasmid] was significantly reduced (Fig. 3C). This result indicated that Sc-p53 could bind to the miR145-5p promoter region to regulate its expression and that the expression of miR145-5p was positively correlated with the expression of Sc-p53 in CPB cells.

### (ii) miR145-5p binding to the c-Myc 3′ UTR.

The result of the MTS assay showed that the concentration of miR145-5p inhibitor and mimics at 50 nM had no evident toxicity for CPB cells (data showed in supplemental material). And reverse transcription-quantitative PCR (qRT-PCR) results showed that compared with the negative control (NC) group, miR145-5p mimics significantly increased miR145-5p expression and decreased c-Myc expression, while miR145-5p inhibitor had opposite effects (Fig. 3D and F). Western blotting results of c-Myc were consistent with the transcription results (Fig. 3E). These results indicated that miR145-5p could negatively regulate c-Myc expression. To assess whether miR145-5p suppressed c-Myc expression by directly targeting its 3′ untranslated region (UTR), a 3,907-bp cDNA sequence of c-Myc was obtained by RACE-PCR. And the potential miR145-5p binding sites were predicted within the 3′ UTR of c-Myc by the RNAhybrid website (Fig. 3G). As shown in Fig. 3H, the luciferase activity of the c-Myc wild-type (WT) 3′ UTR was reduced by miR145-5p mimics and increased by miR145-5p inhibitor, whereas activities of the MUT constructs were not changed. This further demonstrated that miR145-5p could negatively regulate the expression of c-Myc by binding to the 3′ UTR.

### Effects of regulating the Sc-p53/miR145-5p/c-Myc pathway on GLS1.

**FIG 4:**
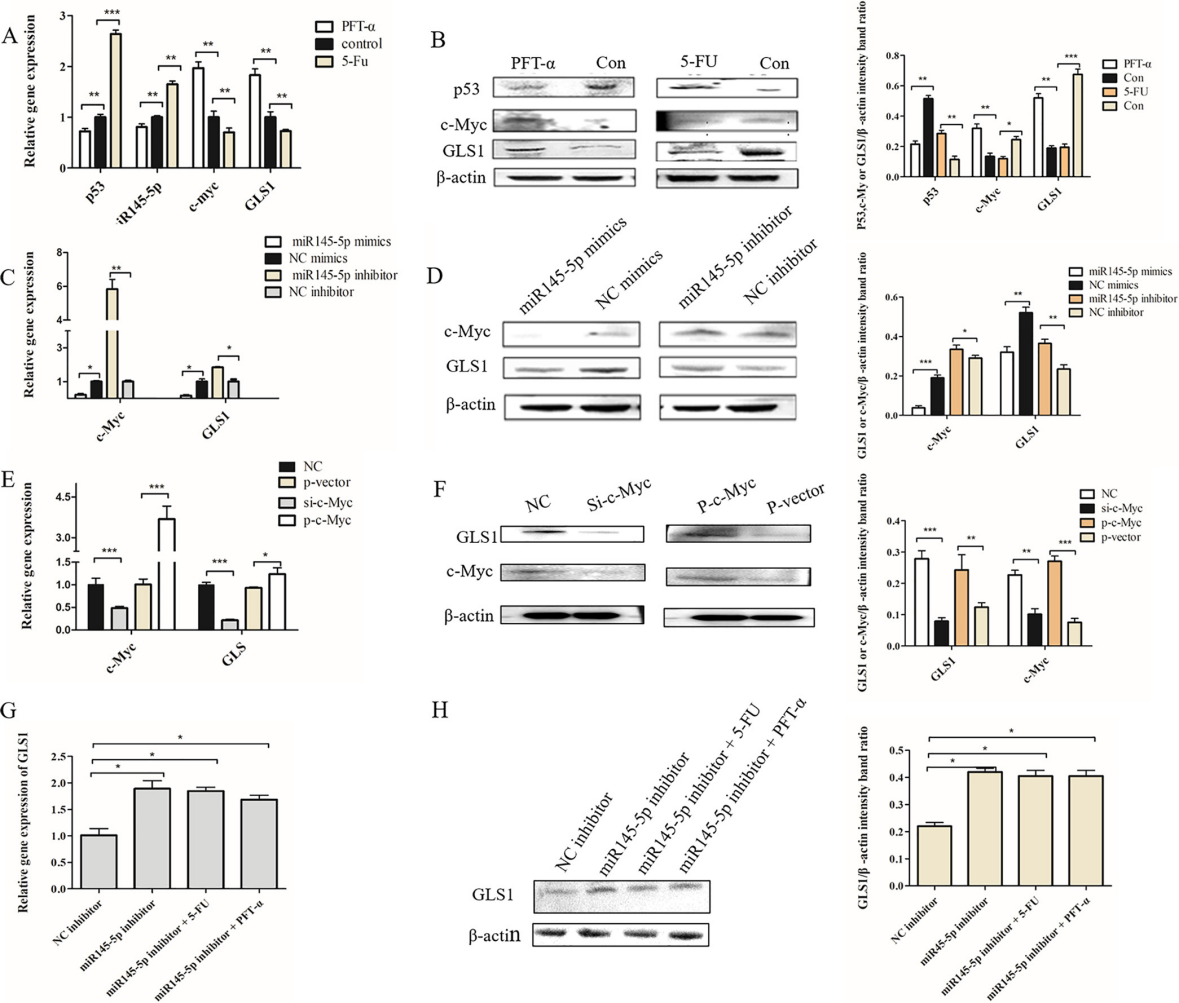
Effects of regulating the Sc-p53/miR145-5p/c-Myc pathway on GLS1. (A and B) The mRNA expression (A) and protein expression (B) of p53, miR145-5p, c-Myc, and GLS1 in CPB cells treated with PFT-α or 5-Fu. (C and D) mRNA (C) and protein (D) expression of c-Myc and GLS1 in CPB cells treated with mimics or inhibitor of miR145-5p. (E and F) mRNA (E) and protein (F) expression of c-Myc and GLS1 in CPB cells transfected with NC small interfering RNA (siRNA) or c-Myc siRNA (si-c-Myc) or pCMV-EGFP vector (p-vector) or pCMV-EGFP-Sc-c-Myc overexpression vector (p-c-Myc). (G and H) mRNA (G) and protein (H) expression of GLS1 in miR145-5p inhibitor-transfected cells treated with PFT-α or 5-Fu. β-Actin was used as a control for qRT-PCR and Western blotting analysis. Asterisks indicate the significant differences from the control group (*, *P* < 0.05; **, *P* < 0.01; ***, *P* < 0.001).

As shown in Fig. 4A and B, compared with the control group, the expression levels of Sc-p53 and miR145-5p were downregulated but c-Myc and GLS1 were upregulated in the PFT-α treatment group. But in the 5-Fu treatment group, the opposite result was observed. Furthermore, the expressions of c-Myc and GLS1 were upregulated after the expression of miR145-5p was inhibited, but the opposite result was observed after miR145-5p was overexpressed (Fig. 4C and D). The results indicated that Sc-p53 could negatively regulate the expression of c-Myc and GLS1 by mediating the expression of miR145-5p.

As shown in Fig. 4E and F, the expressions of c-Myc and GLS1 were decreased after knocking down c-Myc but the expressions of c-Myc and GLS1 were increased after overexpressing c-Myc. This result indicated that the expression of c-Myc was positively correlated with the expression of GLS1. To further verify the regulation between the Sc-p53/miR145-5p/c-Myc pathway and GLS1 expression, the changes of GLS1 expression were investigated after inhibiting the expression of miR145-5p and regulating the expression of Sc-p53 simultaneously. The results showed that there were no differences in GLS1 expression between the miR145-5p inhibitor group and the miR145-5p inhibitor with the 5-Fu or PFT-α groups (Fig. 4G and H). The above results demonstrated that Sc-p53 could regulate GLS1 expression via the Sc-p53/miR145-5p/c-Myc signaling pathway, further influencing the glutaminolysis (see Fig. 6).

### ISKNV and SCRV infection altered the Sc-p53/miR145-5p/c-Myc pathway.

**FIG 5:**
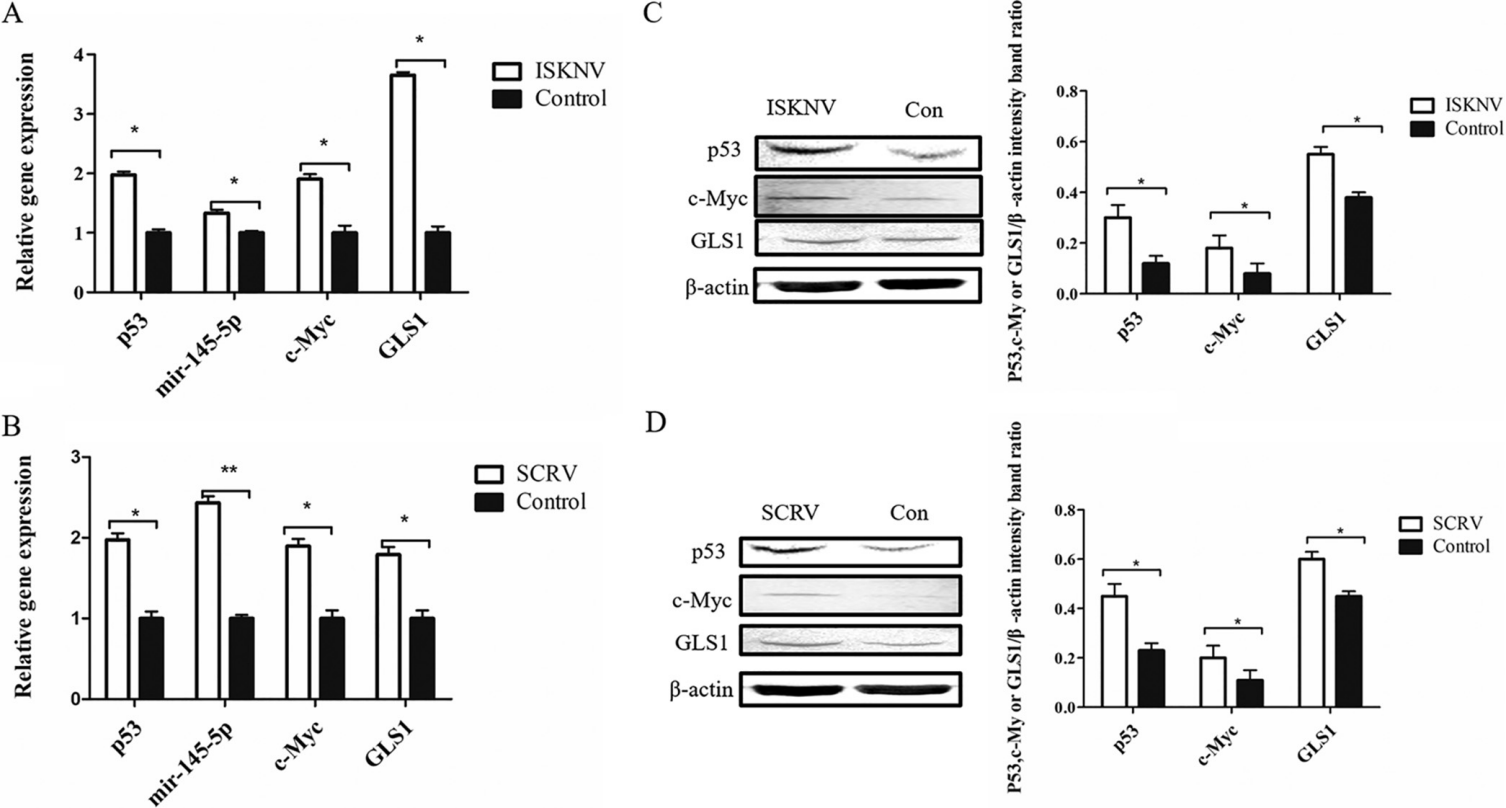
Effects of ISKNV and SCRV infection on Sc-p53/miR145-5p/c-Myc pathway. (A and B) Expression levels of p53, miR145-5p, c-Myc, and GLS1 in CPB cells after ISKNV or SCRV infection by RT-qPCR. (C and D) Western blotting of p53, c-Myc, and GLS1 proteins in CPB cells after ISKNV or SCRV infection. β-Actin was used as a control for qRT-PCR and Western blotting analysis. Asterisks indicate the significant differences from the control group (*, *P* < 0.05; **, *P* < 0.01).

As shown in Fig. 5, compared with the control group, the expressions of p53, miR145-5p, c-Myc, and GLS1 were significantly upregulated after ISKNV and SCRV infection. This result was inconsistent with normal cells, in which miR145-5p negatively regulates the expression of c-Myc and GLS1. Thus, it could be possible that ISKNV and SCRV infection altered the Sc-p53/miR145-5p/c-Myc pathway.

## DISCUSSION

As a transcriptional activator, p53 plays an important role in promoting apoptosis or senescence and cell cycle arrest in response to oncogenic signals (26). Previous studies found that p53 could be implicated in host antiviral defenses (27, 28), such as those against African swine fever virus (ASFV) (29), poliovirus (30), and vesicular stomatitis virus (VSV) (31). These results indicated that Sc-p53 can be used as a target for antiviral research. In the present study, we found that promotion of Sc-p53 expression could also inhibit ISKNV and SCRV proliferation.

Increasing studies have suggested that glutaminolysis plays a significant role in viral replication, and glutamine is required for glutaminolysis (13, 32). Chambers et al. reported that during the course of HCMV infection, the infected cells became dependent upon glutaminolysis for ATP production and viral production; therefore, the level of glutamine consumption increased (33). ATP produced by glutaminolysis is required for the assembly, maturation, and budding of a number of enveloped viruses (34). Our previous studies showed that glutaminolysis was required for efficient replication of ISKNV and SCRV as well, and ISKNV and SCRV replication could be inhibited by inhibiting the expression of enzymes which could affect glutaminolysis, such as GLS, GDH, and IDH2 (12, 25). In the present study, it was shown that the expression of Sc-p53 is negatively correlated with GLS1; accordingly, the glutaminolysis pathway was negatively correlated with the expression of Sc-p53. This result suggested that Sc-p53 was involved in viral replication by regulating the glutaminolysis pathway.

**FIG 6:**
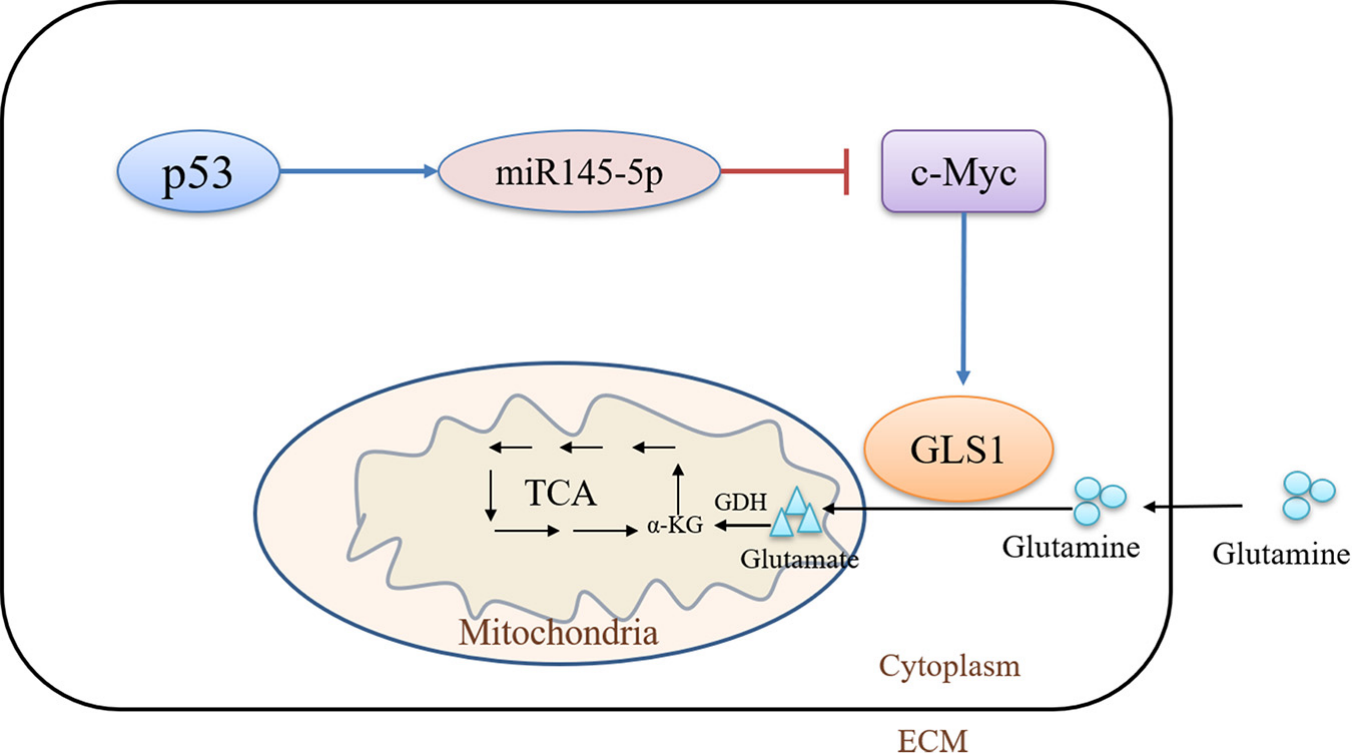
Model of p53 regulation of the glutaminolysis pathway. TCA, tricarboxylic acid; ECM, extracellular matrix.

GLS1 is a key initiation enzyme in the glutaminolysis pathway and plays an important role in glutamine metabolism (15, 35). Previous studies showed that the expression of GLS1 could be regulated by c-Myc through miRNA-23a and mi-RNA23b (17), and it has been reported that p53 could negatively regulate c-Myc through the induction of *miR-145* (21). Our previous studies indicated that Sc-p53 protein was highly similar to p53 proteins of other species (24), suggesting the function of Sc-p53 was similar to that of p53 in other animals. Therefore, it was speculated that Sc-p53 probably regulates GLS1 by regulating the expression of c-Myc via the Sc-p53/miR145-5p/c-Myc pathway. In this study, the Sc-p53/miR145-5p/c-Myc signaling pathway was confirmed by the dual-luciferase reporter system. Sc-p53 could promote the expression of miR145-5p by binding to the miR145-5p promoter region, and miR145-5p could suppress the expression of c-Myc by binding to the c-Myc 3′ UTR. Thus, Sc-p53 could downregulate/upregulate the expression of c-Myc by regulating the expression of miR145-5p and further downregulate/upregulate the expression of GLS1 (Fig. 6). All of these results indicated that Sc-p53 was negatively correlated with the expression of GLS1 via the p53/miR145-5p/c-Myc pathway. However, the expressions of Sc-p53, miR145, c-Myc, and GLS1 in CPB cells infected with ISKNV and SCRV were all upregulated, which was inconsistent with normal cells. Therefore, it could be possible that ISKNV and SCRV infection altered the Sc-p53/miR145-5p/c-Myc pathway of the CPB cells to promote virus proliferation. Similar results related to other viruses have been reported in previous studies as well (13, 36, 37). From the above results, it was inferred that c-Myc might play a crucial role during ISKNV and SCRV infection and could be a potential antiviral therapeutic target.

In conclusion, the present study demonstrated that overexpression of Sc-p53 can negatively regulate glutaminolysis via the Sc-p53/miR145-5p/c-Myc signaling pathway to restrict ISKNV and SCRV replication. However, the Sc-p53/miR145-5p/c-Myc pathway was changed after ISKNV and SCRV infection. This result indicated that viruses could change host metabolic pathways to satisfy the needs of replication and proliferation. All of the above results will provide novel insights into ISKNV and SCRV pathogenesis and antiviral treatment strategies.

## MATERIALS AND METHODS

### Cell line and virus strains.

The Chinese perch brain (CPB) cell line originating from a mandarin fish (*Siniperca chuatsi*) brain was established and stored in our laboratory (38). The CPB cells were propagated and maintained at 28°C in Leibovitz’s L-15 medium (Gibco, USA) supplemented with 10% fetal bovine serum (Gibco, USA) (38). The ISKNV-QY and SCRV-QY strains were isolated and stored in our laboratory (39, 40).

### Pharmaceuticals and antibodies.

Pifithrin-α (PFT-α) and 5-fluorouracil (5-Fu) were purchased from MCE (USA). The rabbit anti-p53, GDH, IDH2, and GLS1polyclonal antibodies and the mouse anti-β-actin polyclonal antibody were purchased from Proteintech (Chicago, IL, USA). The mouse anti-ISKNV-MCP monoclonal antibody and the rabbit anti-c-Myc polyclonal antibody were developed and stored in our laboratory. The rabbit anti-SCRV-N polyclonal antibody was donated by Lin Li from Zhongkai University of Agriculture and Engineering. Horseradish peroxidase (HRP)-conjugated goat anti-rabbit or anti-mouse antibodies were purchased from KPL (USA).

### Viral infection and sample collection.

CPB cells were infected with SCRV or ISKNV (multiplicity of infection [MOI] = 1.0). Following 1 h of adsorption at 28°C, the inoculum was removed and the cells were washed twice with Hanks’ balanced salt solution (HBSS) before adding L-15 medium with 2% (vol/vol) fetal bovine serum (FBS). The SCRV-infected, ISKNV-infected, and mock-infected cells were sampled at indicated time points.

### Total RNA extraction and reverse transcription.

Total RNAs from all samples were extracted using TRIzol reagent (Invitrogen) and resuspended in diethyl pyrocarbonate (DEPC)-treated water. The purity of the RNAs was assessed by the 260-/280-nm absorption ratio, and samples with absorption ratios in the range of 1.8 to 2.0 were used in reverse transcription reactions. First-strand cDNA was synthesized from total RNAs using the PrimeScript RT reagent kit with gDNA Eraser (Perfect Real Time) (TaKaRa, Japan) following the manufacturer’s instructions, and the genomic DNA was ruled out by the gDNA Eraser.

### Sequence amplification and analysis.

To obtain the miR145-5p promoter sequence, a specific amplification was carried out using miR145-5p-specific primers miR145-5p-F and miR145-5p-R (Table 1) designed with Primer 5.0 software according to the mandarin fish transcriptome in our laboratory. The miR145-5p promoter sequence was amplified by PCR. The PCR products were sequenced by IGE (China), and the miR145-5p sequence was verified using the Neural Network Promoter website (http://www.fruitfly.org/seq_tools/promoter.html) and Promoter 2.0 Prediction (https://services.healthtech.dtu.dk/service.php?Promoter-2.0). The target gene of miR145-5p and its target sites were predicted using the RNAhybrid website (https://bibiserv.cebitec.uni-bielefeld.de/rnahybrid).

**TABLE 1:** Primers used in this study

Primer name	Sequence (5′–3′)	Application
miR145-5p-F	CTCTGGTCAACTGGGAGTCTGAGAT	miR145-5p promoter cloning
miR145-5p-R	AGAACAGTATTTCCAGGAATCCCCC	
c-MycD-F	CTCAGTCGCAGGATTGGCTTTA	c-Myc core fragment cloning
c-MycD-R	TGTTTGGTTCTAAGTTTGTCCC	
GSP1	GATTACGCCAAGCTTCTCGTCCTCCAGGCCGTAGATACAG	RACE
GSP2	GATTACGCCAAGCTTCTGCCAGAGTTTGATGAAGGACCTC	RACE
NGSP1	GATTACGCCAAGCTTCCTTCTTCAGGATCACCACCTTGGA	RACE
NGSP2	GATTACGCCAAGCTTGGAGTAAGCCCCTGTCCAAGGAGGA	RACE
UPM Long	CTAATACGACTCACTATAGGGCAAGCAGTGGTATCAACGCAGAGT	RACE
UPM short	CTAATACGACTCACTATAGGGC	RACE
Promoter-F	(KpnI) GGGTACCGGGAGTCTGAGATGAAGCACTGTAG	Plasmid constructions
Promoter-R	(NheI) GGCTAGCTAGGTCAAGGGATTCCTGGGAAAAC	
P53-F	(NheI) GGCTAGCATGGAAGAGCAAAGTTTGGACAATC	Plasmid constructions
P53-R	(XbaI) CTCTAGAGTCGCTGTCACTCCTCTCTCCTCTT	
c-Myc 3′ UTR WT-F	(NheI) GGCTAGCTTCGCAGCTAATGGACCAGG	Plasmid constructions
c-Myc 3′ UTR MUT-F	(NheI) GGCTAGCGGGGGGGCGAGGGGGTTGAAGCAGA	Plasmid constructions
c-Myc 3′ UTR-R	(XbaI) CTCTAGATCTGCTCGCTCCGTCCTCCTTTCAA	Plasmid constructions
c-Myc ORF-F	(EcoRI) GAATTCATGTTGCAAAGCTTCGCTC	Plasmid constructions
c-Myc ORF-R	(ApaI) CCCCGGGTTAGCTGCGAAGCTGCT	
18S-F	CATTCGTATTGTGCCGCTAGA	Real-time quantitative PCR
18S-R	CAAATGCTTTCGCTTTGGTC	
SCRVQ-F	GGCCGTCATGGTGGCGAAT	
SCRVQ-R	GGATAAGTGGCCTGAGCTTC	Real-time quantitative PCR
SCRV-probe	AGAACTGCCTTGACTTCGGCTCC	
ISKNVQ-F	CGAGGCCACATCCAACATC	
ISKNVQ-R	CGCCTTTAACGTGGGATATATTG	Real-time quantitative PCR
ISKNV-probe	CACCAAACTGACCGCGGACTCGT	
GLS1Q-F	GGACATGGAGCAGAGGGACT	
GLS1Q-R	GCTGCAGGATGGTGACTACG	Real-time quantitative PCR
p53Q-F	AGATGCAGAACGGCACCAAG	
p53Q-R	TGTCCAGCAACTCCAGACCA	
c-MycQ-F	ACCGCCACCTTCATCCTCTTCC	Real-time quantitative PCR
c-MycQ-R	CGCCGCTCCTCCTCATCCTC	
miR145-5pQF	GTCCAGTTTTCCCAGGAATCCC	
miR145-5pQR	TCCTTCATTCCACCGGAGTCTG	Real-time quantitative PCR
GDHQ-F	AGGTCCGTCACTATGCCGATGC	
GDHQ-R	AGATCCTCCACCAGCTTGTCCTC	
IDH2Q-F	GTCATCAGTGTGGTCACGGTACG	Real-time quantitative PCR
IDH2Q-R	TGGAGATGGACGGAGACGAGATG	

To obtain the internal core fragment of the cDNA sequence of c-Myc, specific amplification was performed using degenerate c-Myc-specific-primers c-MycD-F and c-MycD-R (Table 1) designed based on the mandarin fish transcriptome in our laboratory. Then, based on the core cDNA sequences of c-Myc, 5′ and 3′ rapid amplification of cDNA ends (RACE) was performed to obtain the full length using a SMARTer RACE cDNA amplification kit (Clontech, Mountain View, CA, USA). The PCR products were sequenced by IGE (China).

### MTS assay and glutamine measurement assay.

The cell viability was assessed using the MTS assay according to the CellTiter 96 AQueous One Solution cell proliferation assay (Promega, USA) protocol. Briefly, cells were seeded (5 × 10^4^ cells/well) in 96-well plates and left overnight to settle; once cells were attached, they were washed once with phosphate-buffered saline (PBS) and then fed with fresh medium supplemented with PFT-α or 5-Fu (0, 5, 10, 15, and 20 μM). At 24, 48, 72, and 96 h posttreatment, 20 μL MTS solution was added into each well and incubated for 3 h at 28°C. Then, cell viability was evaluated using an enzyme-linked immunosorbent assay (ELISA) microplate reader at an optical density at 490 nm (OD_490_) (Infinite M200 Pro; Tecan, Switzerland). Cells treated with medium without pharmaceuticals were used as a control.

Glutamine concentration in CPB cells was measured using a glutamine colorimetric assay kit (BioAssay, USA). In essence, CPB cells in 6-well plates treated with PFT-α or 5-Fu and infected with ISKNV or SCRV were collected at 12 or 24 h postinfection (hpi). Then, glutamine concentration of cells was determined according to the manufacturer’s protocols for the kit. Each group consisted of three parallel wells, and those without treatment were used as a control.

### Pharmaceutical treatment experiment.

CPB cells were seeded into 6-well plates. Once the cells reached 80 to 90% confluence, the cells were washed and treated with PFT-α or 5-Fu at the optimal working concentration for pretreatment time and were collected at 36 h and 48 h posttreatment or infected with SCRV or ISKNV (MOI = 1.0). Then, cells or supernatants were collected for qRT-PCR at 12 and 24 or 36 hpi and for Western blotting at 48 hpi.

### Construction of recombinant plasmid and microRNA and transfections.

The pGL4.17 plasmid (HonorGene, China), pmirGLO plasmid (Promega, USA), pCDNA3.1(+) plasmid, and pCMV-EGFP (enhanced green fluorescent protein) plasmid (stored in our laboratory) were used to construct recombinant plasmid pGL4.17-miR145-5p-promoter (pGL4.17-promoter, containing predicted Sc-p53 seed-matching sites), pCDNA3.1(+)-p53, pmirGLO-c-Myc-WT, pmirGLO-c-Myc-MUT, and p-c-Myc (c-Myc overexpression plasmid). The miR145-5p promoter, Sc-p53 ORF, 3′ UTR WT of c-Myc, 3′ UTR MUT of c-Myc, and c-Myc open reading frame (ORF) were amplified by PCR with the primers in Table 1. The PCR products and the plasmid were digested with the same enzymes, and the target fragments were purified, ligated with T4 ligases (TaKaRa, USA), and then transformed into competent Escherichia coli DH5α cells (TransGen Biotech, China). The recombinant plasmid was verified by sequencing at IGE Biotechnology Ltd. (Guangzhou, China).

The miR145-5p mimics, miR145-5p inhibitor, NC mimics, NC inhibitor, si-c-Myc, and si-NC were synthesized by Shanghai GenePharma Co., Ltd. (Table 2). For transfection, the recombinant plasmid and microRNA were transfected into CPB cells using TransIntro EL transfection reagent (TransGen Biotech, China) according to the manufacturer’s protocols. The c-Myc overexpression plasmid (p-c-Myc) was transfected into CPB cells using FuGENE 6 transfection reagent (Promega, USA).

**TABLE 2:** Interfering RNA sequence of Sc-c-Myc

Name	Sequence (5′–3′)	Antisequence (5′–3′)
si-c-Myc	GCAAUCCAAGAGGGACAAATT	UUUGUCCCUCUUGGAUUGCTT
miR145-5p mimics	GUCCAGUUUUCCCAGGAAUCCC	GAUUCCUGGGAAAACUGGACUU
miR145-5p inhibitor	GGGAUUCCUGGGAAAACUGGAC	
si-NC	UUCUCCGAACGUGUCACGUTT	ACGUGACACGUUCGGAGAATT
NC mimics	UUGUACUACACAAAAGUACUG	
NC inhibitor	CAGUACUUUUGUGUAGUACAA	

### Dual-luciferase reporter assay. (i) p53 and miR145-5p.

CPB cells in 6-well plates were cotransfected with pGL4.17-promoter, pCDNA3.1(+)-p53, corresponding empty plasmids, and pRL-TK plasmid using FuGENE 6 transfection reagent. At 24 h posttransfection, the *Renilla* and firefly luciferase activities were measured using a dual-luciferase assay system (Promega, USA) and firefly luciferase activity was normalized to *Renilla* luciferase activity.

### (ii) miR145-5p and c-Myc.

CPB cells were seeded into a 6-well plate for the luciferase assay. After overnight culture, the cells were cotransfected with luciferase reporter plasmid pmirGLO-c-Myc-WT or pmirGLO-c-Myc-MUT plasmid, together with 50 nM miR-145-5p mimic/inhibitor or NC mimic/inhibitor using TransIntro EL transfection reagent according to the manufacturer’s instructions. At 24 h posttransfection, the *Renilla* and firefly luciferase activities were measured, and the data were expressed as relative firefly luciferase activity normalized to *Renilla* luciferase activity. Simultaneously, the cells treated with miR-145-5p mimics/inhibitor were collected and prepared for detection of the c-Myc expression levels by qRT-PCR and Western blotting.

### Quantitative RT-PCR.

To assess gene mRNA level, the total RNAs of cell samples with virus or pharmaceutical treatment were extracted and the cDNAs were synthesized as described above. qPCR was performed using a SYBR green Pro *Taq* HS kit (AG, China) according to the manufacturer’s instructions. The 18S rRNA was used as the internal control. The primers are listed in Table 1. The relative expression ratio was calculated using the threshold cycle (2^−ΔΔ^*^CT^*) method. Reactions of SYBR green were performed in a 20-μL volume, including 10 μL 2× SYBR Premix, 0.8 μL each forward and reverse primer (10 μM), 0.4 μL ROX reference dye (4 μM), and 6 μL DEPC-water and 2 μL cDNA. All reactions were conducted in triplicate, and the cycling parameters were designed according to the instructions. The qPCR methods for detecting ISKNV and SCRV have been constructed in our laboratory, and the ISKNV and SCRV copies were determined by qPCR as described in the previous report (41, 42).

### Western blotting.

The cells were collected and lysed in radioimmunoprecipitation assay (RIPA) buffer with 1 mM phenylmethylsulfonyl fluoride (PMSF). Then, proteins were electrically transferred onto nitrocellulose paper (0.45 mm; Bio-Rad) using a semidry apparatus (Bio-Rad). Blotted membranes were incubated in PBS-Tween (PBST) containing 5% (wt/vol) nonfat milk at 4°C overnight and then incubated with specific primary antibodies to p53 (1:200); GLS1, IDH2, and GDH (1:500); c-Myc (1:100); ISKNV-MCP (1:500); SCRV-N (1:1,000); and β-actin (1:3,000) at room temperature for 3 h. After washing in PBST for 15 min, the membranes were incubated with the secondary antibody (1:5,000) at room temperature for 1 h. The bands were visualized using HRP enhanced chemiluminescence (ECL) (Millipore, USA) according to the manufacturer’s instructions.

### Statistical analysis.

Statistical data were analyzed by one-way analysis of variance (ANOVA) (expressed as mean + standard deviation [SD]). *P* < 0.05 represented the significance level. All statistical analyses were performed using SPSS 13.0 (SPSS, Chicago, IL, USA).

### Data availability.

The 3,907-bp cDNA sequence of c-Myc obtained by RACE-PCR can be found in GenBank under accession no. MN759310.
